# A milestone for the therapeutic EV field: FDA approves Ryoncil, an allogeneic bone marrow-derived mesenchymal stromal cell therapy

**DOI:** 10.20517/evcna.2025.02

**Published:** 2025-03-25

**Authors:** Bernd Giebel

**Affiliations:** Institute for Transfusion Medicine, University Hospital Essen, University of Duisburg-Essen, Essen 45147, Germany.

**Keywords:** Exosomes, extracellular vesicles, EVs, mesenchymal stem cells, mesenchymal stromal cells

## Abstract

Small extracellular vesicles (sEVs) derived from mesenchymal stromal cells (MSCs) hold substantial promise for therapeutic applications, including immune modulation and tissue regeneration. However, challenges such as batch-to-batch variability, donor material diversity, and the lack of standardized potency testing remain significant barriers to clinical translation. The recent U.S. Food and Drug Administration (FDA) approval of Ryoncil (remestemcel-L) for steroid-refractory acute graft-versus-host disease (aGvHD) in pediatric patients represents a crucial milestone for MSC-based therapies, offering also valuable insights for the development of MSC-EV therapies. This approval highlights the critical need to address variability and standardization issues in MSC products. Strategies like immortalizing MSCs and expanding them clonally can improve scalability, consistency, and overcome limitations inherent to cellular MSC therapies. With the FDA’s decision signaling significant progress, optimizing MSC expansion protocols and refining potency testing methods will be crucial for advancing MSC-EVs as a viable therapeutic option, overcoming current challenges, and expanding clinical applications.

## THERAPEUTIC POTENTIAL OF EXTRACELLULAR VESICLES FROM MESENCHYMAL STROMAL CELLS

Extracellular vesicles (EVs) - especially small EVs (sEVs) with diameters of 70-150 nm, including exosomes, sEVs that derive from the endosomal system - have emerged as critical players in intercellular communication^[1]^. These vesicles regulate a wide array of physiological and pathological processes throughout the body^[2]^. Starting with the observation that B cell-derived exosomes can trigger T cell responses^[3]^, sEVs from various cell types have since been shown to play a pivotal role in modulating diverse immune responses.

In the early 2000s, attention turned to EVs from dendritic cells exposed to tumor antigens. These EVs were shown to present tumor-specific antigens and induce antitumor responses in animal models and in two Phase I clinical trials^[4-7]^. However, a Phase II clinical trial conducted between 2012 and 2014 in non-small cell lung cancer patients failed to demonstrate efficacy, possibly due to the absence of an appropriate adjuvant^[8,9]^. This highlighted the challenges of translating promising preclinical findings into successful human therapies.

Around the same time, pioneering research demonstrated that EVs derived from mesenchymal stromal cells (MSCs) could replicate key therapeutic effects of their parent cells. Notably, MSC-derived EVs were shown to alleviate acute kidney injury and enhance recovery following myocardial infarction in preclinical animal models^[10,11]^. These discoveries, followed by the first successful application of MSC-EVs in an otherwise treatment-refractory acute Graft-versus-Host Disease patient (aGvHD)^[12]^, positioned MSC-derived small EVs (MSC-sEVs) as promising candidates for a wide range of clinical applications, including the treatment of inflammatory diseases and tissue regeneration^[13-23]^. However, much like the development of MSCs as cell-based therapies, the translation of MSC-sEVs into clinical use is anticipated to encounter substantial challenges^[24-28]^. Lessons learned from the MSC field could prove invaluable in navigating these obstacles and accelerating the clinical application of MSC-sEVs.

The approval of Ryoncil (remestemcel-L-rknd), an allogeneic bone marrow-derived MSC therapy for treating steroid-refractory aGvHD in pediatric patients aged 2 months and older, marks the culmination of a long and arduous effort to bring MSC-based products to the U.S. market. This milestone holds significant relevance for the MSC-EV field, offering a roadmap for navigating the complex regulatory and translational challenges associated with EV-based therapeutics.

## MSCS - FROM THEIR DISCOVERY TO U.S. MARKET APPROVAL

The story of MSCs as therapeutic agents began in the late 1960s, when Friedenstein and colleagues first identified MSC-like fibroblasts in adult bone marrow^[29,30]^. These cells, capable of adhering to culture surfaces and differentiating into osteogenic, adipogenic, and chondrogenic lineages^[31]^, were initially envisioned as tools for cell replacement therapies. Early studies suggested MSCs could migrate to damaged tissues and differentiate into cell types like neurons or myocardial cells, raising hopes for their use as “off-the-shelf” therapies for conditions including ischemic stroke and myocardial infarction^[32,33]^.

Upon studying the interaction of MSCs with allogeneic immune cells, MSCs soon revealed an unexpected capability: immunomodulation. Instead of being rejected by the immune system, transplanted MSCs suppressed immune cell activity, including the proliferation of CD4 and CD8 T cells, and promoted regulatory T cell formation^[34-36]^. This discovery opened new therapeutic avenues, particularly in treating inflammatory and autoimmune conditions. A landmark case in this field occurred when Katarina Le Blanc at the Karolinska Institute successfully used MSCs derived from a patient’s mother to treat a 9-year-old boy with acute steroid-refractory GvHD^[37]^. This breakthrough, which was published already in 2004, demonstrated MSCs’ potential as immunomodulatory agents, prompting further exploration of their therapeutic applications.

Over the years, more than 1,700 MSC-related clinical trials have been registered, targeting a range of conditions from degenerative diseases to immune disorders (clinicaltrials.gov). An early first Phase III trial (2006-2009) by Osiris Therapeutics evaluated Prochymal, an MSC therapy for steroid-refractory GvHD. While the treatment was found to be safe, its efficacy was comparable to a placebo^[38,39]^. Following Osiris’ acquisition by Mesoblast, the production strategy was enhanced, leading to the development of remestemcel-L. This improved product demonstrated significant promise in a single-arm Phase III trial for pediatric GvHD, showing notable improvement in treated patients^[40,41]^. However, despite these encouraging results, the U.S. Food and Drug Administration (FDA) declined approval of remestemcel-L twice, in 2020 and 2023. In both instances, the FDA cited concerns about Mesoblast’s potency testing strategy, questioning its ability to accurately and reliably measure a product attribute relevant to the intended therapeutic effect. The FDA argued that the defined critical quality attributes (CQAs) did not ensure consistent potency across independent batches^[42]^.

Despite these initial rejections, MSC therapies have achieved notable milestones globally. For instance, Temcell is approved in Japan for GvHD, and Takeda’s Alofisel was authorized in Europe from 2018 to 2024 for treating complex perianal fistulas in Crohn’s disease^[43-46]^.

Seeking U.S. market approval, Takeda conducted a Phase III clinical trial (ADMIRE-CD II), but despite Alofisel (darvadstrocel) patients responding as expected, the placebo group showed a similarly strong response in this study, ultimately failing to demonstrate the treatment’s efficacy^[47]^. As a result, the European Medicines Agency (EMA) withdrew Alofisel’s market authorization on December 13, 2024.

This highlights that successful clinical translation of MSC products depends on more than manufacturing processes and quality control - it also requires well-designed and properly executed clinical trials. While the EMA’s decision represents a setback for the MSC field in Europe, the recent FDA approval of Ryoncil (remestemcel-L-rknd) in the U.S. could be a game-changer.

On December 18, 2024, the FDA approved Ryoncil for treating steroid-refractory acute GvHD in pediatric patients aged two months and older. As the first FDA-approved MSC therapy in the U.S., Ryoncil represents a major milestone, reinforcing the therapeutic potential of MSCs and the FDA’s commitment to advancing safe and effective treatments for refractory conditions.

The journey of MSCs and their derived products, from initial discovery to clinical approval, exemplifies the challenges and triumphs of translating innovative science into transformative medical treatments. With Ryoncil’s approval, a new era in regenerative and immunotherapy may have begun, offering hope to countless patients worldwide.

## INSIGHTS FROM THE MSC FIELD: GUIDING THE ADVANCEMENT OF MSC-EV THERAPIES

Major challenges in translating MSCs into marketable therapies stem from inherent biological and technical limitations. MSCs exhibit finite expansion capacities, limited lifespan, and progressive alterations in their biological properties during *in vitro* culture. These limitations are compounded by clonal selection procedures, which reduce the clonal heterogeneity of MSC populations in unpredictable ways^[48,49]^. Additionally, aging processes lead to further changes in the biological properties of expanded MSCs over time. This issue persists even in MSCs derived from neonatal tissues or generated from induced pluripotent stem cells (iPSCs), which have enhanced expansion potential^[50-53]^. As a result, ensuring therapeutic efficacy across independent passages remains a key challenge in the MSC field.

Addressing these challenges requires not only defining optimal MSC expansion conditions that increase the likelihood of generating MSC products with consistent therapeutic activities, but also identifying CQAs that effectively reflect therapeutic potency. The inherent heterogeneity of MSCs, combined with donor-to-donor variability and limitations in expansion potential, has created significant barriers to widespread clinical application^[54]^. These issues constrain batch sizes, and the need for diverse donor materials in allogeneic, off-the-shelf products further complicates manufacturing processes. While pooling materials from multiple donors can increase batch sizes^[55,56]^, these strategies do not fully mitigate the challenges posed by limited batch sizes, variability in donor materials, and the absence of reliable potency testing methods.

These are precisely the issues that have led the FDA to withhold market authorization for remestemcel-L^[42]^, despite its demonstrated efficacy in a Phase III clinical trial for pediatric aGvHD patients^[40]^. The unresolved challenges related to batch variability, donor material diversity, and the lack of reliable potency testing have prevented its approval. However, the acceptance of Ryoncil shows that feasible solutions are emerging, which are likely to benefit the broader field.

Previously, we explored whether the significant heterogeneity issues in MSC products could be overcome by using their EVs. While we have reported that MSC-EVs show therapeutic activity in various animal models and in a GvHD patient^[12,57-69]^, we have also learned that not all MSC-EV products exhibit activity in preclinical models^[62,64,69]^. Upon further investigation, we observed that even when starting with the same MSC stocks and applying a standardized MSC-EV manufacturing procedure, independent batches derived from aliquots of the same cell stock often differ in their immunomodulatory and therapeutic properties^[69]^. This variability mirrors the same challenges seen in the MSC field.

Drawing from the experience in the MSC field, we recognize that addressing this issue is urgent. To overcome this, we and others have immortalized MSCs and expanded them at the clonal level^[68,70,71]^. Indeed, analyses have shown that the resulting MSC-EV products exhibit high batch-to-batch consistency, addressing some of the challenges related to cell aging and clonal selection. A critical task is to confirm that culture-expanded immortalized MSCs maintain their characteristics over multiple passages and do not undergo changes due to high mutation rates, epigenetic shifts, or developmental processes that could reintroduce MSC heterogeneity. Additionally, we must convincingly demonstrate that the immortalizing effects of the parental cells are not transferred via the EV populations to other cells.

Furthermore, the remestemcel-L case highlights the importance of robust potency testing strategies, which we have discussed in detail elsewhere^[26,27]^. While the MSC-EV field may face additional challenges, the manufacturing of MSC-EVs is fundamentally reliant on MSC expansion processes. With recent progress, we are optimistic that MSC expansion protocols will meet regulatory requirements for market approval, leaving downstream processing in EV product manufacturing as the remaining challenge. Given that well-established techniques used in recombinant antibody production and vaccine manufacturing are already available^[72]^, we are optimistic that these issues can be resolved swiftly. Thus, the latest market approval decisions have provided a critical signal to the MSC-EV field, signaling progress and encouraging further innovation.

## DECLARATIONS

### Copyright

© The Author(s) 2025.
